# The influence of the representation of collagen fibre organisation on the cartilage contact mechanics of the hip joint

**DOI:** 10.1016/j.jbiomech.2016.03.050

**Published:** 2016-06-14

**Authors:** Junyan Li, Xijin Hua, Alison C. Jones, Sophie Williams, Zhongmin Jin, John Fisher, Ruth K. Wilcox

**Affiliations:** a Institute of Medical and Biological Engineering, School of Mechanical Engineering, University of Leeds, UK; b School of Mechanical Engineering, Xi’an Jiaotong University, China

**Keywords:** Contact mechanics, Articular cartilage, Biphasic, Collagen fibre, Finite element

## Abstract

The aim of this study was to develop a finite element (FE) hip model with subject-specific geometry and biphasic cartilage properties. Different levels of detail in the representation of fibre reinforcement were considered to evaluate the feasibility to simplify the complex depth-dependent fibre pattern in the native hip joint. A FE model of a cadaveric hip with subject-specific geometry was constructed through micro-computed-tomography (µCT) imaging. The cartilage was assumed to be biphasic and fibre-reinforced with different levels of detail in the fibre representation. Simulations were performed for heel-strike, mid-stance and toe-off during walking and one-leg-stance over 1500 s. It was found that the required level of detail in fibre representation depends on the parameter of interest. The contact stress of the native hip joint could be realistically predicted by simplifying the fibre representation to being orthogonally reinforced across the whole thickness. To predict the fluid pressure, depth-dependent fibre organisation is needed but specific split-line pattern on the surface of cartilage is not necessary. Both depth-dependent and specific surface fibre orientations are required to simulate the strains.

## 1 Introduction

Finite element (FE) models of the natural hip have the potential to be used to examine how diseases and therapies affect the biomechanical performance of the joint (Henak et al., 2013, Li et al., 2014b). However, there are a number of challenges to the incorporation of the geometrical and material properties with sufficient realism to enable meaningful predictions to be made.

From a geometric perspective, it has been shown that FE models with realistic joint geometry predict different contact mechanics than models with idealised geometry (i.e. spherical joint geometry and uniform cartilage thickness). This is reflected in more irregular distributions of stress and strain and higher magnitudes of contact stress in the more realistic geometry models (Anderson et al., 2010).

From a materials perspective, the biomechanical performance of the joint is closely linked to the biphasic structure of the cartilage (interstitial fluid and solid matrix) as well as to the organisation of collagen fibres embedded within its solid matrix (Mow et al., 1980, Ateshian et al., 1994, Li et al., 2000, Korhonen et al., 2003, Shirazi et al., 2008).

The importance of interstitial fluid within the cartilage to the biomechanical function of the hip joint has been demonstrated in FE models with idealised geometry (Li et al., 2013, Li et al., 2014a), where the cartilage has been represented as a biphasic material. Here it was found that, for short term and dynamic loading, the interstitial fluid within the cartilage supports the majority of the load through pressurisation and only a small portion of load is supported by the solid matrix.

The collagen fibres within the cartilage provide mechanical stiffness and maintain the structure of the solid matrix (Mow et al., 1980, Ateshian et al., 1994). It has been demonstrated that the inclusion of isotropic fibre reinforcement in the cartilage (i.e. different properties in tension and compression) within FE models of the hip greatly alters the predicted contact mechanics (Li et al., 2014c). Through the thickness of healthy cartilage, fibres are distributed in different patterns. The tissue thickness can be divided into three regions: the surface zone in which fibres are parallel to the articulating surface, the middle zone in which fibres are randomly distributed and the deep zone where fibres are perpendicular to the subchondral bone (Weiss et al., 1968, Mow et al., 1992, Buckwalter et al., 1994). The fibre orientation in the surface zone of cartilage can be visualised by inserting a needle dipped in ink into the surface of cartilage and is sometimes referred to as the split-line pattern. In the healthy hip joint, fibres in the surface zone align towards the fossa of acetabulum and femoral head (Mital and Millington, 1970), hypothesised to align with the directions of maximum principal tensile strain (Bullough and Goodfellow, 1968, Armstrong, 1986).

The representation of fibre orientation in the surface zone of cartilage has been demonstrated to play an important role in FE models of the knee as well as at the tissue level (Mononen et al., 2012, Li et al., 2009). However, the effect of the inclusion of more realistic fibre orientation in FE models of the hip has not been previously investigated. One of the main challenges is that the incorporation of zonal differences of fibre pattern into whole joint computational models requires the cartilage to be modelled with a number of elements through the thickness, leading to lengthy pre-processing and processing of the model (Li and Gu, 2011). There is also a need to evaluate the necessity of incorporating the complex depth-dependent fibre pattern in the whole hip joint.

In recent studies, biphasic fibre-reinforced materials have been incorporated into FE models of whole joints (Dabiri and Li, 2013, Halonen et al., 2014, Mattei et al., 2013). However, to achieve numerical convergence, these models were either only loaded to a low level of load (non-physiological) (Mattei et al., 2013, Dabiri and Li, 2013, Halonen et al., 2014) or monophasic materials were used as one of the bearing surfaces (e.g. biphasic cartilage in contact with elastic meniscus in the knee (Mononen et al., 2012, Kłodowski et al., 2015) and hemiarthroplasty in the hip (Pawaskar et al., 2010, Pawaskar et al., 2011)).

Recently, using an open source FE solver specifically designed for biomechanical applications (FEBio, http://febio.org/febio), convergence has been improved in a hip FE model with idealised geometry, enabling physiological loads to be applied (Li et al., 2013, Li et al., 2014a). In a recent study, this method has also been used to simulate a porcine hip hemiarthroplasty model in which both biphasic cartilage and realistic joint geometry were considered (Li et al., 2014c). However, biphasic fibre-reinforced properties have yet to be incorporated into 3D whole hip models with subject-specific geometry under physiological loading.

The aim of this study was to evaluate the effect of implementing different levels of detail in the fibre reinforcement within the articular cartilage of a hip FE model. Specifically, the objectives were to examine the need for realistic split-line representation of fibre orientation in the surface layer, and for implementing different fibre patterns through the cartilage thickness. To this end, a FE model of the hip with subject-specific geometry incorporating biphasic fibre-reinforced cartilage properties that could be solved under both physiological and prolonged loading was developed. The effect of the different levels of detail in the fibre representation was examined in terms of differences in the predicted contact mechanics and cartilage strain states.

## 2 Methods

### 2.1 Imaging, segmentation and solid model construction

**Fig. 1:**
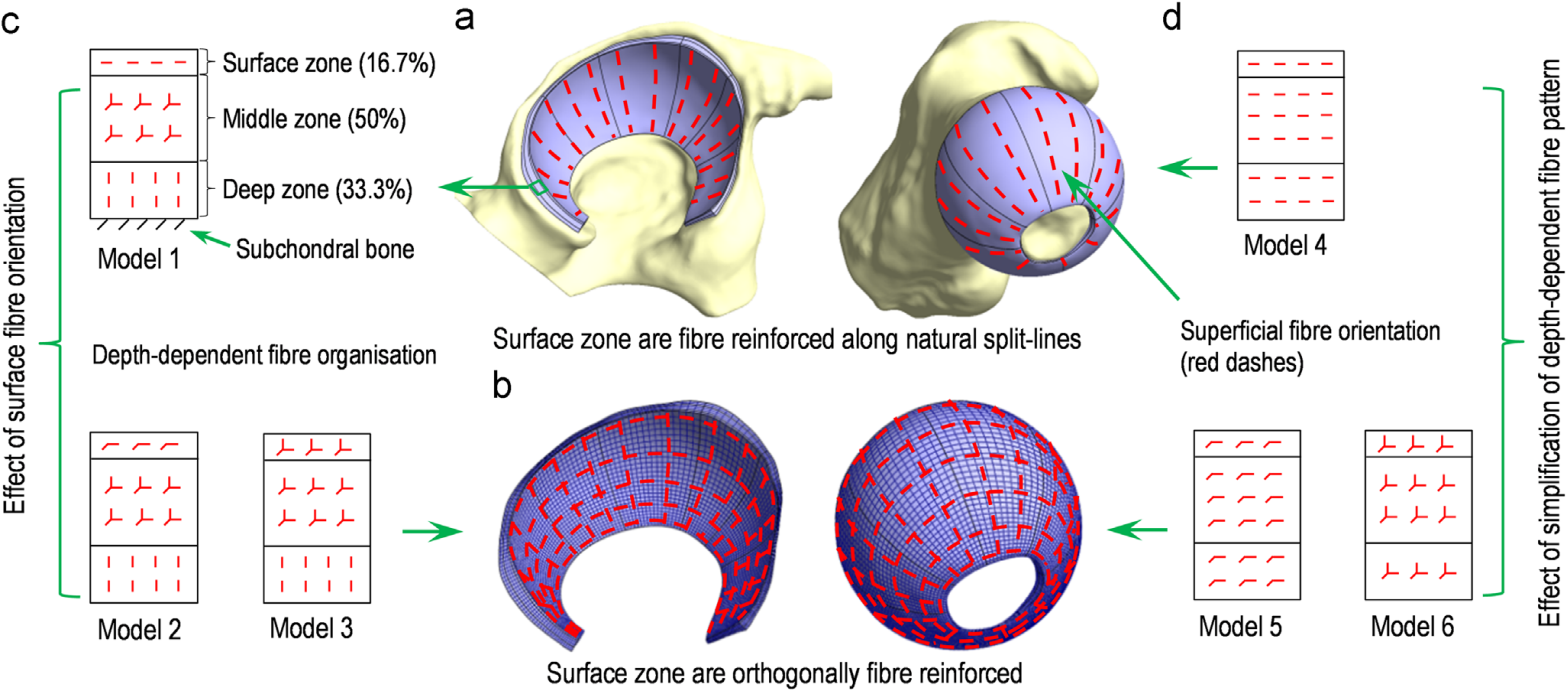
Creation of FE models with different fibre patterns. Models 1, 2 and 3 had depth-dependent fibre reinforcement pattern (c). Fibres were assumed uniformly distributed in all directions in the middle zone and perpendicular to the subchondral bones surface in the deep zone. Fibre reinforcement in the surface zone of Models 1, 2 and 3 had different orientations: along a single direction following the natural split-line pattern in Model 1; orthogonally across the articulating surfaces in Model 2; uniformly distributed in all spatial directions in Model 3. Models 4, 5 and 6 had a uniform fibre pattern through the thickness (d) with all the fibres oriented in a single direction following the natural split-line pattern in Model 4; orthogonal fibre distribution parallel to the articulating surfaces in Model 5 and uniform fibre distribution in all spatial directions in Model 6.

A femur and a pelvis from a 55 year-old 109 kg male at the time of death (cause of death: alcoholic cirrhosis of the liver) was adopted in this study. Non-transplantable human cadaveric tissue was supplied by Platinum Training and its use approved by the University of Leeds research ethics committee. The position of femur and pelvis were recorded based on anatomical landmarks (Bergmann et al., 2001). The specimen was then dissected to retain the hip region. A subject-specific FE model of this hip specimen was constructed and simulated using a validated process described previously (Li et al., 2014c). Briefly, the femoral and acetabular components were imaged separately using a micro-computed-tomography (µCT) scanner (μCT 80, SCANCO Medical AG, Brüttisellen, Switzerland) at a cubic voxel size of 73.6 µm and energy of 70 kVp, 114 µA, providing good visualisation of the bone and cartilage. The volumetric µCT data were segmented and smoothed in an image processing software package (ScanIP version 5.1; Simpleware Ltd., Exeter UK) and then exported into Geomagic Studio (version 11, Geomagic Inc., Research Triangle Park, NC, USA) to construct the solid model (Fig. 1a) which was meshed in ABAQUS (version 6.11-1, Dassault Systemes, Suresnes Cedex, France) (Fig. 1b).

### 2.2 FE model construction and material properties

To facilitate the incorporation of the pattern of collagen fibres in the surface zone (i.e. aligned towards the fossa of the acetabulum and femoral head (Mital and Millington, 1970) (Fig. 1a)), the solid model of the cartilage was partitioned along the split-lines so that the meshed elements and fibres could be oriented along these directions (Maas et al., 2012). The model was meshed with six layers of elements through the cartilage thickness. It has been reported that in healthy cartilage, the surface, middle and deep zones account for 10–20%, 40–60% and 30–40% of the thickness (Mow et al., 1992, Buckwalter et al., 1994), and were therefore represented by one, three and two layers of elements respectively (Fig. 1c). The femoral head cartilage and acetabular cartilage were represented with about 28,000 and 24,000 eight-noded hexahedral elements (hex8, FEBio User Manual 1.7, Section 3.5.2.1 “Solid Elements”) respectively. The mesh density was selected such that a change of less than 5% difference in contact stress and fluid pressure occurred if the number of elements were doubled.

The cartilage was assumed to be biphasic with a fibre-reinforced solid matrix. Fibre reinforcement was incorporated into the cartilage model to provide linear elastic tensile stiffness but no resistance for the tissue in compression (coefficient of exponent *α*, power of exponent *β* and fibre modulus ξ (*α*=0 and *β*=2 for the discontinuous transition from compression to tension), fibre-reinforced direction controlled by *θ* and *φ*, FEBio User Manual 1.7, section 4.1.3.6 “Fibre with Exponential-Power Law”, febio.org/febio/febio-documentation). Considering the much smaller dimension of collagen fibres than the elements in the model, the uniformly distributed fibres, as seen in the middle zone of the healthy cartilage, were represented by incorporating fibre reinforcement along three orthogonal directions so that each element was isotropically reinforced (Fig. 1c). To evaluate the influence of fibre pattern in the surface zone on the joint biomechanics, three models with depth-dependent and different surface fibre patterns were generated (Models 1, 2 and 3 in Fig. 1c). In addition, three other models with uniform zonal fibre pattern (Models 4, 5 and 6) were generated in order to evaluate the effect of simplifying the fibre pattern as being uniform through the cartilage thickness (Fig. 1d). The Young modulus, Poisson’s ratio and permeability of the non-fibrillar matrix were assumed to be 1.2 MPa, 0.045 and 0.0009 mm^4^/Ns respectively (Athanasiou et al., 1994). The fibre modulus of the cartilage with space-orthogonally reinforced fibre pattern was set to 10 times higher than the non-fibrillar modulus (Cohen et al., 1998, Soltz and Ateshian, 2000, Li et al., 2014c). The same fibrillar density was assumed for all the models and for all the three cartilage zones in order to eliminate the effect of fibre stiffness (Table 1).

### 2.3 Boundary conditions and simulation

The bone was assumed to be rigid (Li et al., 2013), and the surfaces of the cartilage of the acetabulum and femoral head that were connected to the subchondral bone were rigidly constrained to two reference points respectively. Through the reference point, the acetabular cartilage was fixed in all the degrees of freedom and the femoral head cartilage was constrained in rotational degrees of freedom but free to move translationally. A force was applied to the femoral head through the reference point to match the magnitude and spatial direction of the in vivo hip contact forces measured by Bergmann et al. (2001) (kinematics and kinetics data provided in Hip98 (Bergmann, 2001)). Due to the lengthy period of simulating a whole gait cycle, loading at heel-strike, mid-stance and toe-off during walking were applied separately and ramped over 1 s. The variation in loading period of these different gait phases was not considered, because the time-dependent behaviour of the hip cartilage has been found to be minimal over short periods of loading (Li et al., 2013, Li et al., 2014a). In addition, to simulate one-leg-stance over a prolonged period, loading was applied over 1 s and then held constant for 1500 s to evaluate the time-dependent biomechanical performance of the models. For each loading scenario, the femur and pelvis were repositioned based on the anatomical landmarks and kinematics data. The loading was first normalised to bodyweight (BW) and then scaled to the BW of the donor of the specimen used in this study: heel-strike: 264% BW (2820 N); mid-stance: 166% BW (1773 N); toe-off: 199% BW (2126 N); one-leg-stance: 243% BW (2596 N). The contact between the articulating surfaces was assumed to be frictionless. Fluid flow through the articulating surfaces was contact dependent, i.e. fluid was allowed to flow between contacting surfaces as well as from open surfaces of the cartilage (Maas et al., 2012, Li et al., 2013).

All analyses were conducted using the open-source non-linear FE solver FEBio (version 1.8; http://febio.org/febio) due to its ability to achieve convergence when dealing with biphasic materials in contact. The models were solved on a Linux server with 8 GB of RAM and 8 Intel X5560 cores at 2.8 GHz. Contact stress (i.e. compressive stress of the cartilage surface in the direction perpendicular to the articulating surfaces), fluid pressure, solid matrix compressive stress (the contact stress minus the fluid pressure (Mow et al., 1980)) in the cartilage surface, maximum and minimum Lagrange strains and maximum principal stress were recorded.

The data associated with this paper are openly available from the University of Leeds Data Repository (10.5518/49).

## 3 Results

**Fig. 2:**
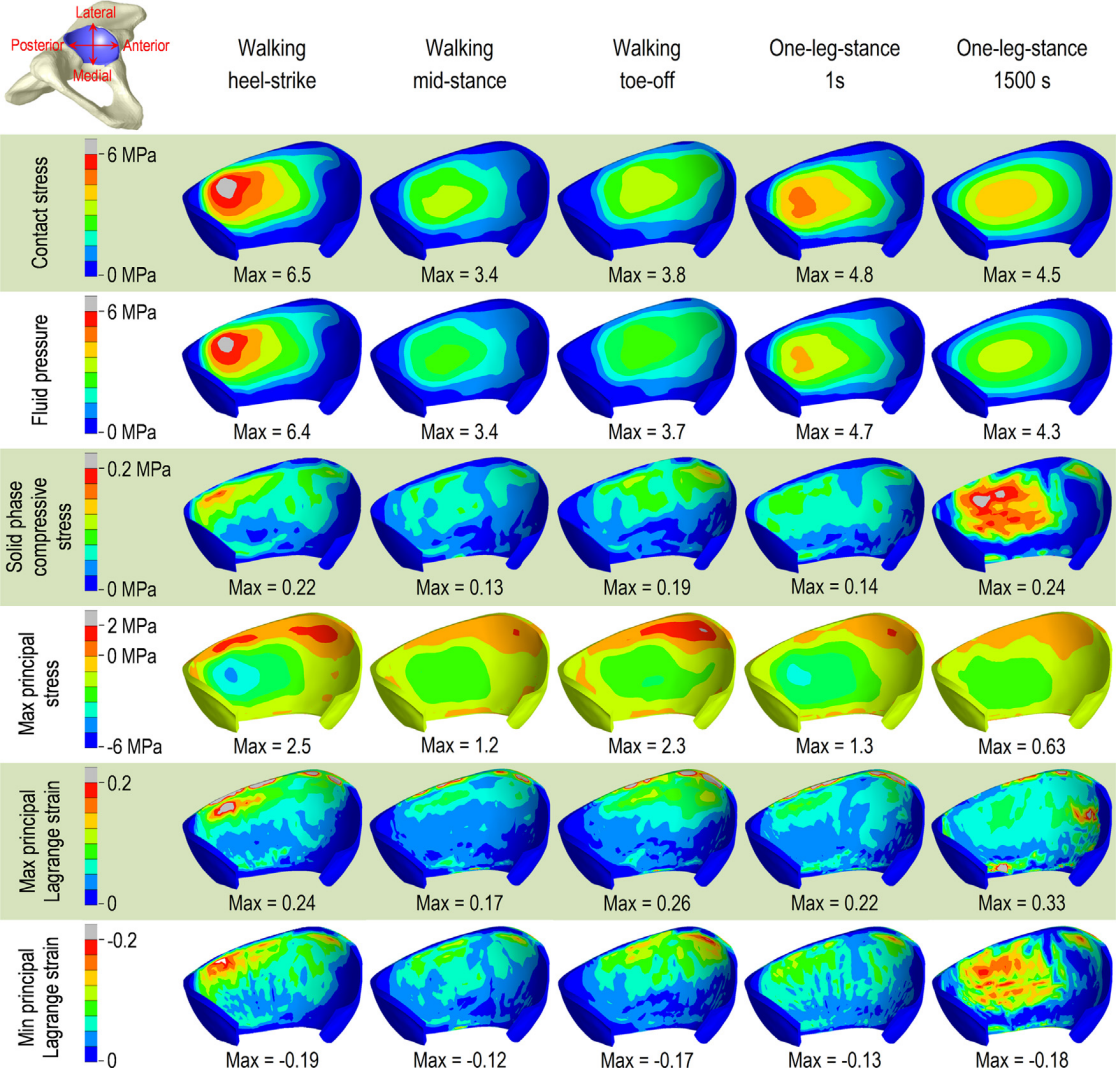
Contours of results on the surface of the acetabular cartilage in Model 1. Contact stress and fluid pressure contours on the cartilage surface were similar. Peak contact stress occurred at heel-strike and was markedly greater than compressive stress in the solid phase of the cartilage. Peak strains and maximum principal stresses occurred in the posterolateral edge of the acetabular cartilage at heel-strike and in the anterolateral edge at toe-off. Solid phase compressive stress and minimum principal strain increased over the 1500 s of one-legged stance. Positive values of maximum principal stress denote the region of cartilage in tension, while negative value indicate compression along all the principal directions.

As shown in Fig. 2, the contact stress and fluid pressure contours on the acetabular cartilage surface were similar in Model 1. The solid phase compressive stress was less than 10% of the contact stress. The peak contact stress occurred at heel-strike. The maximum and minimum principal strains and maximum principal stress were concentrated in the posterolateral edge of the acetabular cartilage at heel-strike and slid towards the anterolateral edge at toe-off. Over 1500 s, the peak solid phase compressive stress and minimum principal strain were increased by 71% and 38% respectively.

**Fig. 3:**
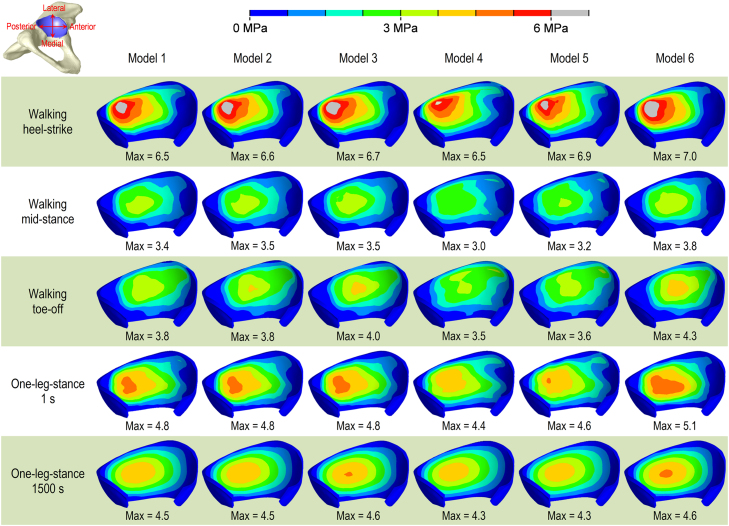
Contours of the contact stress for the models with different fibre organisations. The magnitude and distribution of contact stress were similar among the models.

Comparable magnitudes and distributions of contact stress were observed for all the models (less than 15% difference) (Fig. 3). In all the models, the contact stress altered in distribution and slightly decreased in magnitude over 1500 s. Similar results were also observed for the fluid pressure on the surface of cartilage.

**Fig. 4:**
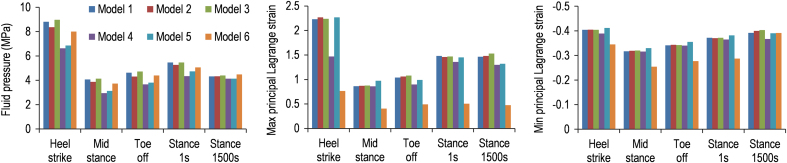
Comparison of peak results in the whole cartilage of Models 1–6. Models 1, 2 and 3 had similar peak maximum and minimum principal strains (difference less than 5%). Model 1 had more similar strains to Model 5 than Models 4 and 6. Differences in the peak maximum and minimum principal strains in the whole cartilage between Models 1 and 5 were less than 15%, while the peak fluid pressure of Model 5 was over 20% lower than Model 1 during heel-strike and mid-stance.

In the whole cartilage (Fig. 4), Models 1, 2 and 3 had similar peak maximum and minimum principal strains (difference less than 5%). Model 1 had more similar strains to Model 5 than Models 4 and 6. Differences in the peak maximum and minimum principal strains in the whole cartilage between Models 1 and 5 were less than 15%, while the peak fluid pressure of Model 5 was over 20% lower than Model 1 during heel-strike and mid-stance.

**Fig. 5:**
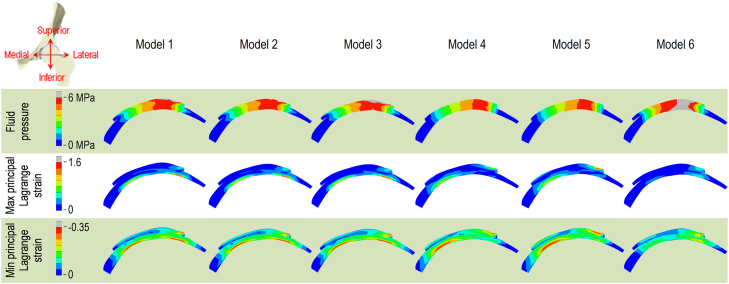
Cross-section of the cartilage of all the models, showing fluid pressure and strains through the depth of the cartilage. The fluid pressure was less depth-dependent compared to the strains. Compared to Model 1, Models 2 and 3 had more similar cross-sectional distribution of fluid pressure and strains than Models 4, 5 and 6.

Through the depth of the cartilage (Fig. 5), the fluid pressure was less depth-dependent compared to the strains. Compared to Model 1, Models 2 and 3 had more similar cross-sectional distribution of fluid pressure and strains than Models 4, 5 and 6.

**Fig. 6:**
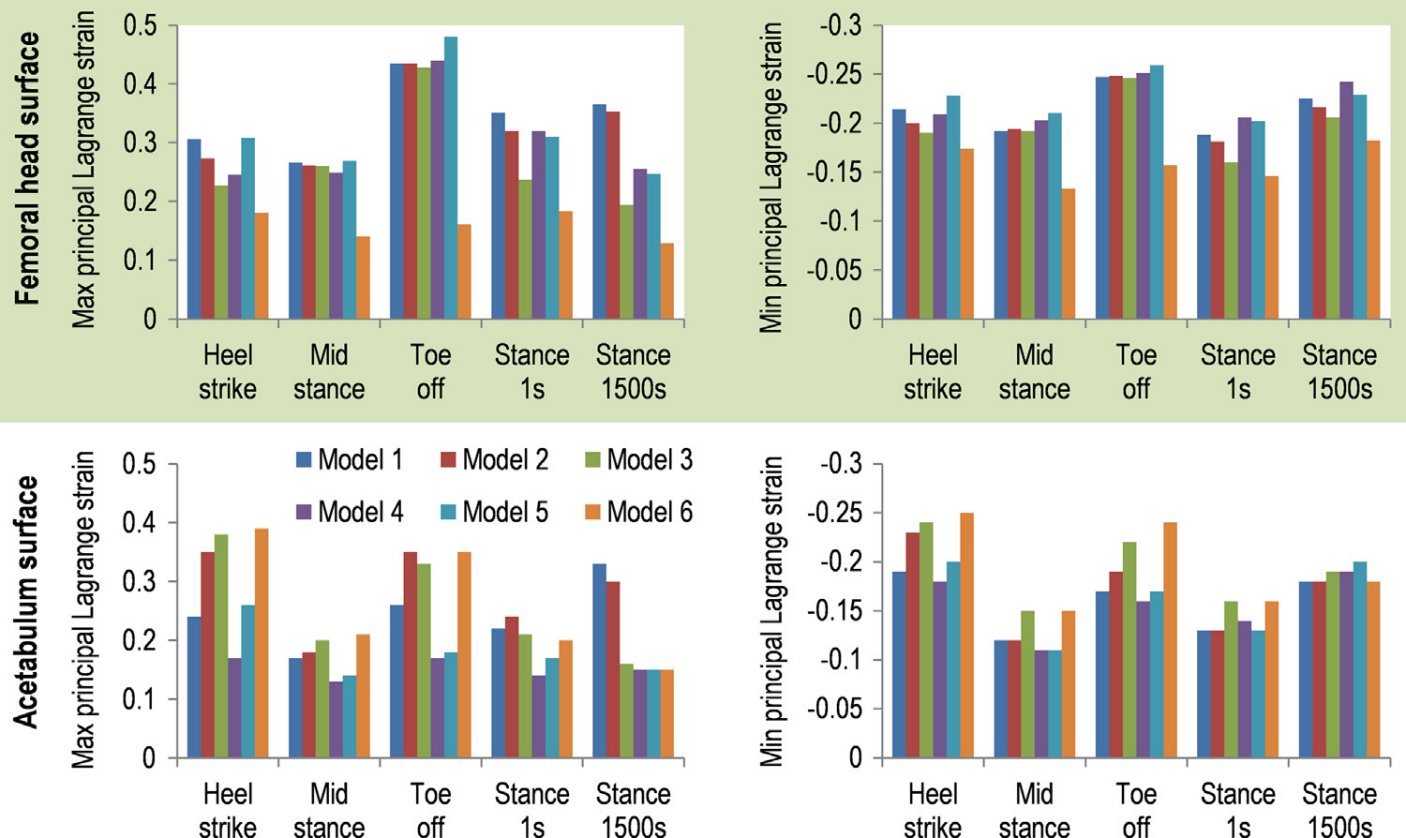
Comparison of peak maximum and minimum principal Lagrange strains on the surface of the acetabular and femoral head cartilage in Models 1–6. The peak strains on the cartilage surface of Model 1 were higher on the femoral side and lower on the acetabular side than Models 2 and 3. The variation in the strains among Models 1, 2 and 3 was generally greater in the acetabular side than the femoral side. The magnitude of difference in the peak strains between Model 1 and Models 2–6 was over 30%.

On the surface of the cartilage (Fig. 6), Model 1 had higher peak maximum and minimum principal strains in the femoral side than Models 2 and 3. However, on the acetabular cartilage surface, the peak strains of Model 1 were lower than Models 2 and 3. The variation in the strains among Models 1, 2 and 3 was generally greater in the acetabular side than the femoral side. For example, the maximum principal strain of Model 3 at heel-strike was 58% higher on the acetabular cartilage surface and 26% lower on the femoral head cartilage surface than Model 1. The magnitude of difference in the peak strains between Model 1 and Models 2–6 was over 30%.

## 4 Discussion

In this study, a full hip FE model with subject-specific geometry and biphasic cartilage properties was developed and simulated under physiological and prolonged loading conditions for the first time. Different representations of the fibre reinforcement within the cartilage were simulated in the model. The model without fibres (i.e. solid matrix with homogenous isotropic properties) was not considered in this study, because it has been previously demonstrated that removal of fibre network from the hip cartilage leads to greatly altered joint mechanics (Li et al., 2014c).

The less than 6% difference in contact stress between Models 1 and 5 suggested that it is feasible to improve computational efficiency through reducing the number of elements to predict hip contact stress, where this is the output of interest. Fluid pressure in Model 1 was similar to Models 2 and 3 but different from Models 4, 5 and 6. Therefore, to predict fluid pressure, depth-dependent fibre organisation is needed but specific split-line pattern on the surface of cartilage is not necessary. However, over 30% differences in the strains were observed between Model 1 and Models 2–6, suggesting that both depth-dependent fibre pattern and native surface fibre orientation is needed for predictions of these variables.

In a previous study, the biphasic properties of cartilage were successfully incorporated into a hip model with generic geometry (Li et al., 2013) and subject-specific geometry was also considered in a biphasic porcine hemiarthroplasty model (Li et al., 2014c). In the current study, the methodology was improved to incorporate subject-specific geometry of the whole hip, specifically with non-uniform shape and thickness of the cartilage, which has been shown to provide more realistic predictions of the joint biomechanics (Anderson et al., 2010). A major challenge in solving biphasic whole joint models is the high computational expense, particularly in the case of a prolonged loading period. In this study, the simulation period for model applied with a load over 1500 s was up to four weeks. One reason for the lengthy simulation period was the large number of elements because the depth-dependent fibre organisation required at least six layers of elements through the thickness of the cartilage.

When the hip was loaded, the acetabular cartilage was compressed towards the lateral edge of the acetabulum where large strains occurred, due to the very congruent joint geometry and the horseshoe shape of acetabular cartilage (Fig. 4, Fig. 5). The natural fibre orientation in the surface layer helped prevent the cartilage from being overly stretched towards the lateral edge of acetabulum and thus decreased the magnitude of strains. This is demonstrated by the lower strains on the acetabular cartilage surface of Model 1, compared to Models 2 and 3. These results suggested that fibres in the surface layer were orientated along the direction in which they can effectively resist the strain of the acetabular cartilage. The variation in results among Models 1, 2 and 3 was generally greater in the acetabular side than the femoral side, suggesting that surface fibre orientation plays a more important role in constraining strains in the horseshoe-shaped acetabular cartilage than the ball-shaped femoral head cartilage. The contribution of realistic fibre orientation to controlling strain in the cartilage was also found in a previous knee model (Mononen et al., 2012). In contrast, the contact stress, fluid pressure and compressive stress in the solid matrix were similar between the models with different fibre patterns, potentially because the same fibre density was assumed for all the models. This suggests that the fibre orientation in the surface zone of cartilage had minor influence on these parameters, which is also consistent with the findings in a previous knee model (Mononen et al., 2012).

The maximum contact stress of Model 1 during walking is 6.5 MPa under a load of 2820 N which is consistent with previous experimental measurements (4–10 MPa under loads of 2500 N–3000 N) (Brown and Shaw, 1983, Afoke et al., 1987, Anderson et al., 2008). The magnitude of minimal principal strain was 0.1–0.2 in the surface zone of cartilage and 0.3–0.4 in the whole cartilage. This is also in good agreement with previous studies in which the magnitude of minimal principal strain is approximately 0.1 on the surface of cartilage (Mononen et al., 2012) and ranges from 0.3–0.5 through the whole cartilage region (Ferguson et al., 2000, Greaves et al., 2010).

Under instantaneous loads, subject-specific joint geometry has been shown to have a great influence on the contact mechanics of the hip in a previous hip model with elastic cartilage (Anderson et al., 2010, Harris et al., 2012). Over a prolonged loading period, the biphasic model with realistic joint geometry in this study had distinctly faster cartilage consolidation process as well as markedly higher magnitude and more irregular distribution in contact stress as compared to the previous biphasic model with generic geometry (Li et al., 2013), demonstrating that the subject-specific joint geometry also has a great influence on the time-dependent biomechanical performance of the hip.

There were some limitations to this study. First, the labrum was excluded because it was difficult to differentiate it from other soft tissues in the µCT images (i.e. ligaments and membrane) and there is limited reported data on its multi-phasic material properties. The incorporation of the labrum into the biphasic model would also considerably increase the computational expense due to the large number of elements that would be required to represent its irregular geometry. Although the labrum has been found to provide little assistance in load bearing of the hip (Henak et al., 2011) and confining the cartilage strain (Greaves et al., 2010), the labrum does help impede the fluid exudation process (Ferguson et al., 2000, Ferguson et al., 2003) and thus may decelerate the cartilage consolidation process over the 1500 s loading period in this study. Ligaments were not included because contribution of ligament forces was taken into account in the joint contact force applied. Another limitation is that the material properties in this study were adopted from the literature. Fibre properties were assumed to have discontinuous tension–compression transition, and the strain-dependent nonlinear fibre modulus as reported in other cartilage models (Shirazi et al., 2008) were not considered. Additionally, depth-dependent changes in cartilage properties were not considered. However, these assumptions do not affect the qualitative predictions of this study, i.e. biomechanical differences found between models with different fibre organisations. Nevertheless, the need for validated specimen-specific material properties is a consideration in future subject-specific modelling studies. The time-dependent response of the hip joint is minimal during the first several cycles of walking (Li et al., 2014a), but whole gait loading would be important if a prolonged walking period is of interest. Although the modelling was based on the previously validated procedure of a hemiarthroplasty hip, the entire hip biphasic model has not been validated to date due to the challenges in measuring time-dependent contact pressure in the very congruent geometry of the hip.

In conclusion, a new full hip FE model with subject-specific geometry and biphasic fibre-reinforced cartilage properties that can be applied with physiological and prolonged loads was developed to evaluate different representations of the fibre reinforcement. It was found that the level of detail in fibre representation depends on the parameter of interest. The contact stress of the native hip joint could be realistically predicted by simplifying the fibre representation to being orthogonally reinforced across the whole thickness (i.e. Model 5). To predict the fluid pressure, depth-dependent fibre organisation is needed but specific split-line pattern on the surface of cartilage is not necessary. Both depth-dependent and specific surface fibre orientations are required to simulate the strains. This study provides the methodological platform to investigate biomechanical changes caused by degeneration as well as potential interventions in the future.

## Conflict of interest

None of the authors have any financial or personal relationships with other people or organisations that could have inappropriately influenced or biased the work.

## Figures and Tables

**Table 1:** Fibre orientation and modulus. The same fibrillar density was assumed for the models with different fibre organisations.

**Symbol**	**Fibre reinforcement orientation**	**Fibrillar modulus (MPa)**
	Only one direction parallel to articulating surface	36
	Only one direction perpendicular to subchondral bone	36
	Two orthogonal directions parallel to articulating surface	18
	Orthogonally in all three spatial directions	12
